# Implementation of triage in a pediatric emergency department

**DOI:** 10.1186/1865-1380-7-S1-O3

**Published:** 2014-07-25

**Authors:** Uma Priya Narayanan, Swathi Padankatti, Kuruvilla Thomas

**Affiliations:** 1 Sundaram Medical Foundation Dr Rangarajan Memorial Hospital, Shanthi Colony, Anna Nagar, Chennai, India

## Objective

To implement SATS (South African Triage system) in a pediatric emergency department.

## Methods

Design: Prospective observational study

Setting: Pediatric Emergency Department (ED) of a Community Hospital with 10500 ED visits annually. The hospital conducts DNB Pediatrics, MCEM and BSc Accident & Emergency Technology courses.

Participants: 3693 children between 0 and 18 years of age who attended ED in a six month period from September 2011 to February 2012

Tools: Nurses on triage duty applied SATS to all children attending ED during the study period; data was compiled and analysed.

SATS is a validated four-category color-coded triage system. Rapid evaluation of clinical discriminators and an age-appropriate composite physiological score called Triage Early Warning Score (TEWS) constitute triage. Points are given for normal versus abnormal mobility, respiratory rate, heart rate, temperature and presence or absence of trauma. TEWS has three versions - below 3 years, 3-12 years and above 12 years.

## Results

Of 3693 triaged children, 74 (2%), 469 (12.7%), 1054 (28.6%), 2096 (56.9%) were in the emergency (red), very urgent (orange), urgent (yellow) and non-urgent (green) groups respectively. 3299 children were discharged from the ED with 50 (1.51%), 383 (11.6%), 939 (28.4%), 1927 (58.4%) from red, orange, yellow, green groups respectively. Of 372 hospitalized children, 16 (4.3%), 76 (20.4%), 112 (29.7%), 168 (45.1%) were in red, orange, yellow, green groups respectively. 22 were referred to other hospitals; there were no deaths.

50 of red and 383 of orange group were discharged from ED; over-triage rate was 26%. 161 of green group were hospitalized; under-triage rate was 7.6%.

Mean time for triage was 4.4 minutes (range 3-5 minutes).

## Limitations

Inter-observer variation in assigning triage codes was not studied; effectiveness of triage would depend on experience of the Emergency nurse. SATS was not compared with other triage tools.

## Conclusion

Our study supports the use of SATS as a primary triage tool in pediatric ED. Under- and over-triage rates were within the limits of ACSCOT guidelines. Percentage over-triage was higher than under-triage, thereby erring on the safe side.

